# MRI radiomics to monitor therapeutic outcome of sorafenib plus IHA transcatheter NK cell combination therapy in hepatocellular carcinoma

**DOI:** 10.1186/s12967-024-04873-w

**Published:** 2024-01-19

**Authors:** Guangbo Yu, Zigeng Zhang, Aydin Eresen, Qiaoming Hou, Emilie Elizabeth Garcia, Zeyang Yu, Nadine Abi-Jaoudeh, Vahid Yaghmai, Zhuoli Zhang

**Affiliations:** 1 Department of Biomedical Engineering, University of California Irvine, Irvine, CA USA; 2 Department of Radiological Sciences, School of Medicine, University of California Irvine, 839 Health Sciences Rd., Irvine, CA 92617 USA; 3 Department of Chemistry, Physical Science, University of California Irvine, Irvine, CA USA; 4 Information School, University of Washington, Seattle, WA USA; 5 Chao Family Comprehensive Cancer Center, University of California Irvine, Irvine, CA USA; 6 Department of Pathology and Laboratory Medicine, University of California Irvine, Irvine, CA USA

**Keywords:** Hepatocellular carcinoma (HCC), Sorafenib, Natural killer cell, Immunotherapy, Radiomics, Magnetic resonance imaging (MRI)

## Abstract

**Background:**

Hepatocellular carcinoma (HCC) is a common liver malignancy with limited treatment options. Previous studies expressed the potential synergy of sorafenib and NK cell immunotherapy as a promising approach against HCC. MRI is commonly used to assess response of HCC to therapy. However, traditional MRI-based metrics for treatment efficacy are inadequate for capturing complex changes in the tumor microenvironment, especially with immunotherapy. In this study, we investigated potent MRI radiomics analysis to non-invasively assess early responses to combined sorafenib and NK cell therapy in a HCC rat model, aiming to predict multiple treatment outcomes and optimize HCC treatment evaluations.

**Methods:**

Sprague Dawley (SD) rats underwent tumor implantation with the N1-S1 cell line. Tumor progression and treatment efficacy were assessed using MRI following NK cell immunotherapy and sorafenib administration. Radiomics features were extracted, processed, and selected from both T1w and T2w MRI images. The quantitative models were developed to predict treatment outcomes and their performances were evaluated with area under the receiver operating characteristic (AUROC) curve. Additionally, multivariable linear regression models were constructed to determine the correlation between MRI radiomics and histology, aiming for a noninvasive evaluation of tumor biomarkers. These models were evaluated using root-mean-squared-error (RMSE) and the Spearman correlation coefficient.

**Results:**

A total of 743 radiomics features were extracted from T1w and T2w MRI data separately. Subsequently, a feature selection process was conducted to identify a subset of five features for modeling. For therapeutic prediction, four classification models were developed. Support vector machine (SVM) model, utilizing combined T1w + T2w MRI data, achieved 96% accuracy and an AUROC of 1.00 in differentiating the control and treatment groups. For multi-class treatment outcome prediction, Linear regression model attained 85% accuracy and an AUC of 0.93. Histological analysis showed that combination therapy of NK cell and sorafenib had the lowest tumor cell viability and the highest NK cell activity. Correlation analyses between MRI features and histological biomarkers indicated robust relationships (r = 0.94).

**Conclusions:**

Our study underscored the significant potential of texture-based MRI imaging features in the early assessment of multiple HCC treatment outcomes.

**Supplementary Information:**

The online version contains supplementary material available at 10.1186/s12967-024-04873-w.

## Background

Hepatocellular carcinoma (HCC) is the most common primary liver malignancy and the third leading cause of cancer-related deaths worldwide [1]. Surgical resection and liver transplantation serve as potentially curative treatments. However, only a small percentage of patients are eligible for surgery [2]. For patients with unresectable HCC, loco-regional therapies and systemic treatments are the primary therapeutic options, with limited success in improving overall survival [3]. Sorafenib, a multi-targeted kinase inhibitor targeting various protein kinases, is an FDA-approved systemic treatment option for hepatocellular carcinoma (HCC) [4]. It serves as a crucial therapeutic option, working by inhibiting serine-threonine kinases Raf-1 and B-Raf, as well as the receptor tyrosine kinases of VEGFR 1, 2, 3 and PDGFR-β, thereby preventing tumor cell proliferation and angiogenesis [5]. However, sorafenib is associated with various side effects, which may require dose reductions or treatment discontinuation [6].

In the current therapeutic environment, natural killer (NK) cell-based adoptive immunotherapy (NK-ATI) has shown promise in the treatment of several cancers, including HCC [7]. Despite its considerable potential, NK-ATI treatments have demonstrated limited therapeutic efficacy. These therapies' drawbacks include a lack of NK cytotoxicity function, insufficient NK cell homing to tumors, and a lack of early noninvasive techniques for predicting NK-ATI response [8, 9]. The combination of sorafenib and NK cells has been shown to prime proinflammatory responses of tumor-associated macrophages (TAMs) within the HCC microenvironment, perpetuating cytotoxic NK cell activity, and increasing populations of total NK and CD56^dim^ NK cells in peripheral blood, demonstrating the potential of this combined therapy [10, 11].

Magnetic resonance imaging (MRI) plays an essential role in cancer treatment evaluations, offering a noninvasive method to assess treatment efficacy. Historically, cancer treatment outcomes have relied on traditional metrics like tumor size changes. However, these metrics may not comprehensively capture intricate tumor alterations, particularly with immunotherapy [12]. Cancer immunotherapy can exhibit distinctive response patterns, such as transient tumor swelling (pseudoprogression), delayed regression, and new lesions [13]. Consequently, conventional size-based criteria like RECIST 1.1 might fall short in therapeutic response evaluations [14]. Hence, advanced methods leveraging tumor tissue characteristics are essential for accurate therapeutic response determination.

Recent advancements in medical imaging have shown that texture analysis— an advanced method of extracting tissue characteristics from conventional medical images— proves essential in diagnosing conditions, gauging disease severity, and predicting patient survival in clinical oncology settings [15–17]. However, studies exploring predictive models using MRI texture features for combined drug delivery and immunotherapy effects remain limited.

In oncology, discerning multi-class treatment outcomes, rather than simple binary predictions, presents a complex challenge. This study uses magnetic resonance imaging (MRI) and texture analysis based on machine learning techniques to conduct a comprehensive and noninvasive assessment of therapeutic response. The primary objective of our study is to investigate the initial treatment response of a combined therapy using sorafenib and NK cells in a hepatocellular carcinoma (HCC) rat model.

This study aimed to predict therapeutic outcomes following combined sorafenib and intrahepatic arterial transcatheter engineered NK cells tumor-targeted delivery for HCC treatment using conventional MR images with radiomics analysis.

## Materials and methods

### Cell lines and cell culture

The N1-S1 hepatoma cell line (CRL-1604, American Type Culture Collection, Manassas, VA) was cultured in Iscove's Modified Dulbecco’s Medium (IMDM) supplemented with 10% fetal bovine serum (FBS), 1.25% GlutaMAX (Gibco, Waltham, MA), and 1% penicillin/streptomycin (Gibco, Waltham, MA). Cells were maintained at 37 °C in 5% CO_2_ and 95% air, subcultured every three days, and monitored for viability (> 90%) using a Countess II automated cell counter (Life Technologies, Carlsbad, CA) with 0.4% trypan blue dye. The RNK-16 rat NK cell line, provided by Thomas L. Olson (University of Virginia, Charlottesville, VA), was cultured in RPMI medium with the addition of 25 mM 2-Mercaptoethanol. Before experimental use, RNK-16 cells were treated with recombinant mouse IL-12 and IL-18 for 24 h, followed by PBS washing and resting in a fresh medium for an additional 24 h.

### Tumor cell implantation

Animal studies were conducted following protocols approved by our Institutional Animal Care and Use Committee. Twenty-four Sprague Dawley (SD) rats (Charles River Laboratories, Hollister, CA), weighing between 250 and 300 g (6–8 weeks old), underwent subcapsular implantation of 1 × 10^6^ N1-S1 cells into the left lateral lobe of the liver under 2% isoflurane anesthesia. Post-operative care included administration of buprenorphine and meloxicam for pain relief. Animals were observed daily for signs of distress, and tumors were allowed to grow before initiation of therapeutic intervention.

### Therapeutic approaches: NK cell immunotherapy and sorafenib treatment

Once the tumors had reached a size of 5 mm under MRI guidance, the animals were randomly assigned into four distinct groups: NK cell immunotherapy, sorafenib treatment, combined NK cell, sorafenib administration, and control group (n = 6 per group). Sorafenib administration involved daily gastric gavage of a 10 mg/kg dose for seven days. For the NK cell and combined therapy groups, 1 × 10^7^ RNK-16 cells were delivered via the intrahepatic arterial (IHA) catheter following the procedure described by Sheu et al. [18]. All procedures and animal observations in accordance with institutional ethical guidelines.

### MRI acquisition

The animals were imaged with a 3T MRI scanner (Philips Achieva, Best, Netherlands) and commercial wrist coil. Anesthesia was delivered using 1–2% isoflurane inhalation at a rate of 2 L/min. MRI scans were conducted weekly for up to 2 weeks post-treatment to monitor tumor progression and assess treatment efficacy in vivo. The MRI sequences included (a) axial T2w MRI with settings of repetition time (TR): 3500 ms, echo time (TE): 63.177 ms, slice thickness (ST): 2 mm (no gap), flip angle (FA): 90º, field‐of‐view (FOV): 50 × 50 mm^2^, and number of signals averaged (NSA): 4; (b) T1w FFE with TR: 200 ms, TE: 2.45 ms, ST: 2 mm (no gap), FA: 90º, FOV: 50 × 50 mm^2^, and NSA: 4. The ITK-SNAP (v.4.0) software was used for the delineation of tumor regions in the T1w and T2w MRI images, which were then applied to all acquired images following an affine transformation [19].

### Histology analysis

Upon study termination, animals were humanely euthanized in accordance with IACUC regulations. Tumor-bearing liver were excised, and 4 mm-thick tissue blocks centrally encompassing tumors were immediately fixed in 10% formalin. The specimens were then paraffin-embedded and sectioned (5 µm thick). Histological staining involved hematoxylin and eosin (H&E) to assess tumor viability and CD56^+^ antibody labeling to assess NK cell viability. These procedures were facilitated by the University of California Irvine Experimental Tissue Shared Resource Facility (Orange, CA).

Histology slides were digitized using the Hamamatsu whole slide scanner and analyzed with QuPath (v0.4.3) and ImageJ (v.1.54c). We quantified the percentage of viable tumor cells from five representative sections, chosen randomly and observed at 100 × magnification, stained with H&E dye. Blinded researchers then quantified viable tumor cells and NK cells from five randomly selected regions at 100 × magnification. NK cell migration was assessed on CD56^+^-stained slides by calculating the proportion of positively stained cells relative to the total, taken from five random fields at 100 × magnification.

### Feature extraction

MRI Texture features were extracted using PyRadiomics (version 3.1.0, PyRadiomics Community) [20]. Quantitative features of tumor tissues were extracted from designated regions of interest (ROIs) using seven methods: first-order statistics (FoS), shape-based features (SP), gray level co-occurrence matrix (GLCM), gray level run length matrix (GLRLM), gray level size zone matrix (GLSZM), gray level dependence matrix (GLDM), and neighborhood gray tone difference matrix (NGTDM). Additionally, three filters, laplacian of gaussian (LoG), gradient and wavelet transform (WT), were applied to enhance feature extraction. To eliminate the effects caused by tumor size, features linked to this variable, including voxel number, axis length, maximum diameter, mesh surface, and pixel surface, were excluded.

Subsequently, texture features were computed from the filtered images, resulting in a total of 743 texture features extracted from the T1w and T2w MRI data separately. These features were then standardized using z-score normalization to offset the relative intensity variations inherent in MRI data. To minimize multicollinearity, Pearson correlation was utilized to evaluate cross-correlation coefficients. Features exhibiting a strong correlation (|r|> 0.8) were removed from the set. Afterward, we employed the recursive feature elimination (RFECV) algorithm with cross-validation, leveraging the support vector machines (SVM) classification model, to further reduce features number. To ensure a 10:1 sample size to feature ratio, we further refined our selection by choosing the top five features based on their importance rankings in a random forest model for our subsequent analyses.

### MRI radiomics classifications

Based on the optimal feature subset, we constructed four classification models: support vector machine (SVM), XGBoost, random forest (RF) and linear regression model (LR). We employed fivefold cross-validation combined with grid search to identify the optimal hyperparameters for each model. The chosen hyperparameters maximized the area under the curve (AUC) of the receiver operating characteristic (ROC) curve. Leave one out cross-validation (LOOCV) was used to leverage all the data during model training. The performance of the classification models was measured with accuracy, area under the receiver operating characteristics curve (AUC), sensitivity, and specificity. For binary classification models, considering the imbalance between groups, the synthetic minority oversampling (SMOTE) algorithm was used to balance the number of treatment and control group samples. For multi-class classification models, ROC curves using micro-averaged one-vs-the-rest (OvR) were used to assess the model’s performance.

### Statistical analysis

**Fig. 1:**
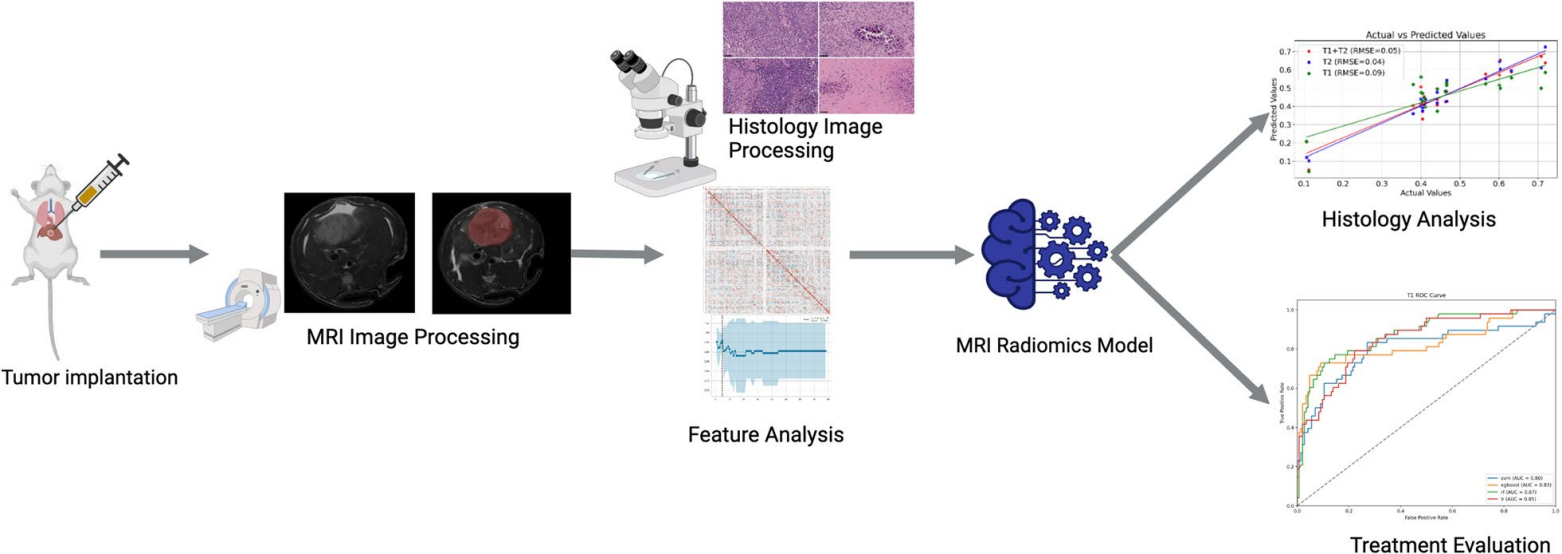
Flowchart summarizes the tumor treatment evaluation process. The process begins with tumor implantation in a rat model, followed by the acquisition and processing of MRI images. Subsequently, radiomics features are extracted and fed into the MRI radiomics model. This model is designed to predict HCC treatment outcomes and establish correlations with histological biomarkers

To correlate MRI manifestations of HCC treatment outcomes with changes identified through histological tumor markers, we developed three multivariable linear regression models using quantitative features from T1w, T2w, and combined T1w + T2w MRI datasets. Model performance was evaluated using root-mean-squared-error (RMSE) and Spearman correlation coefficient. The analysis was conducted using Scikit-learn in Python (Fig. 1).

## Results

### Feature selection

**Fig. 2:**
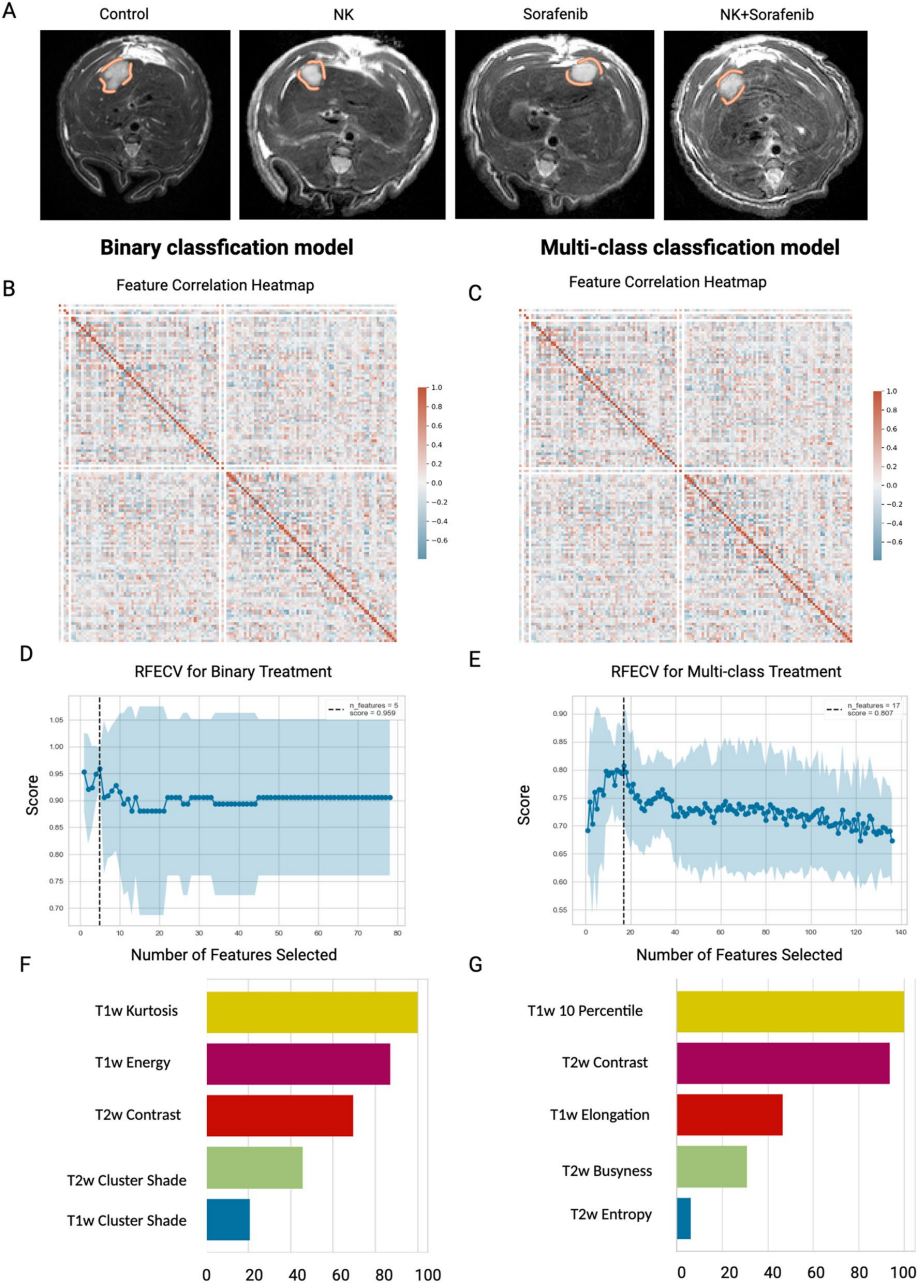
Feature selection process for T1w + T2w MRI data (**A**) Baseline MRI representative images of rat treatment groups, with a circle highlighting the tumor region (**B**) Feature correlation heatmap for binary classification model (**C**) Feature correlation heatmap for multi-class classification model (**D**) RFECV feature selection process for binary classification model (**E**) RFECV feature selection process for multi-class classification model (**F**) Features importance

From T1w and T2W MRI data, 743 features were derived using seven distinct feature extraction methodologies and three filtering techniques. Following a collinearity analysis, features exhibiting high correlation were removed, thus reducing the feature set to 80. This subset included 3 shape-based features (SP), 27 FoS, 23 GLCM features, 21 GLRLM features, 5 NGTDM features, and 1 GLDM feature. The inter-feature correlation among these 80 features was visualized using a heatmap, as presented in Fig. 2B, C. Subsequently, the feature set underwent a further feature selection process employing RFECV algorithm, combined with SVM classification model and evaluated based on AUROC score (Fig. 2D, E). The final features chosen for the modeling are shown in Table 1.

**Table 1:** The list of features used to generate classification models for HCC treatment efficacy

Task	T1w	T2w	T1w + T2w
Binary treatment	Energy (FoS)	Contrast (NGTDM)	T1w Kurtosis (FoS)
	Kurtosis (FoS)	Energy (FoS)	T1w Energy (FoS)
	Contrast (NGTDM)	Kurtosis (FoS)	T2w Contrast (NGTDM)
	Elongation (Shape)	Maximum (Original)	T2w Cluster Shade (GLCM)
	Contrast (GLCM)	Kurtosis (FoS)	T1w Cluster Shade (NGTDM)
Multi-class treatment	10 Percentile (FOS)	Contrast (NGTDM)	T1w 10 Percentile (FoS)
	Kurtosis (FoS)	Perimeter Surface Ratio (Shape)	T2w Contrast (NGTDM)
	Minimum (FoS)	Mean (Original)	T1w Elongation (Shape)
	Contrast (NGTDM)	Elongation (Shape)	T2w Busyness (NGTDM)
	Minimum (LoG filtered FOS)	Long Run Low Gray Level Emphasis (GLRLM)	T2w Entropy (FoS)

### Evaluation of the binary classification models

**Fig. 3:**
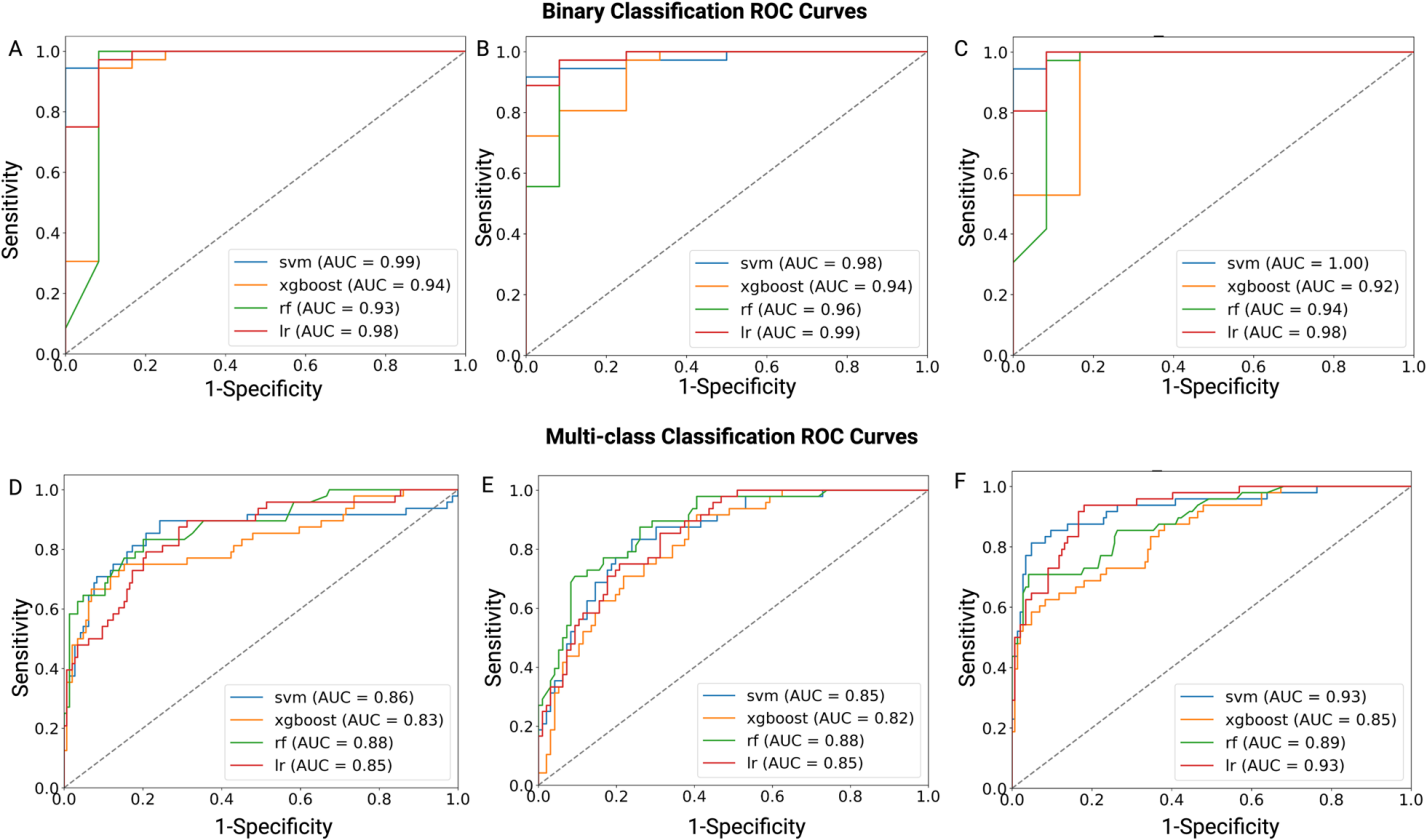
Receiver operating characteristic (ROC) curves for evaluating HCC treatment responses. **A**, **C** ROC curves illustrating the discriminative performance of four models—SVM, XGBoost, RF, and LR—in differentiating between the treatment and control groups. (**A**) uses T1w MRI data, (**B**) uses T2w MRI data, and (**C**) uses a combined T1w + T2w MRI dataset. **D**–**F** ROC curves showing the classification abilities of the same models across various treatment groups based on (**D**) T1w MRI, (**E**) T2w MRI, and (**F**) combined T1w + T2w MRI datasets. These groups include the control group (no treatment), sorafenib-only treatment, NK cell infusion via hepatic artery (IHA), and a combination therapy of sorafenib and NK cell IHA

**Fig. 4:**
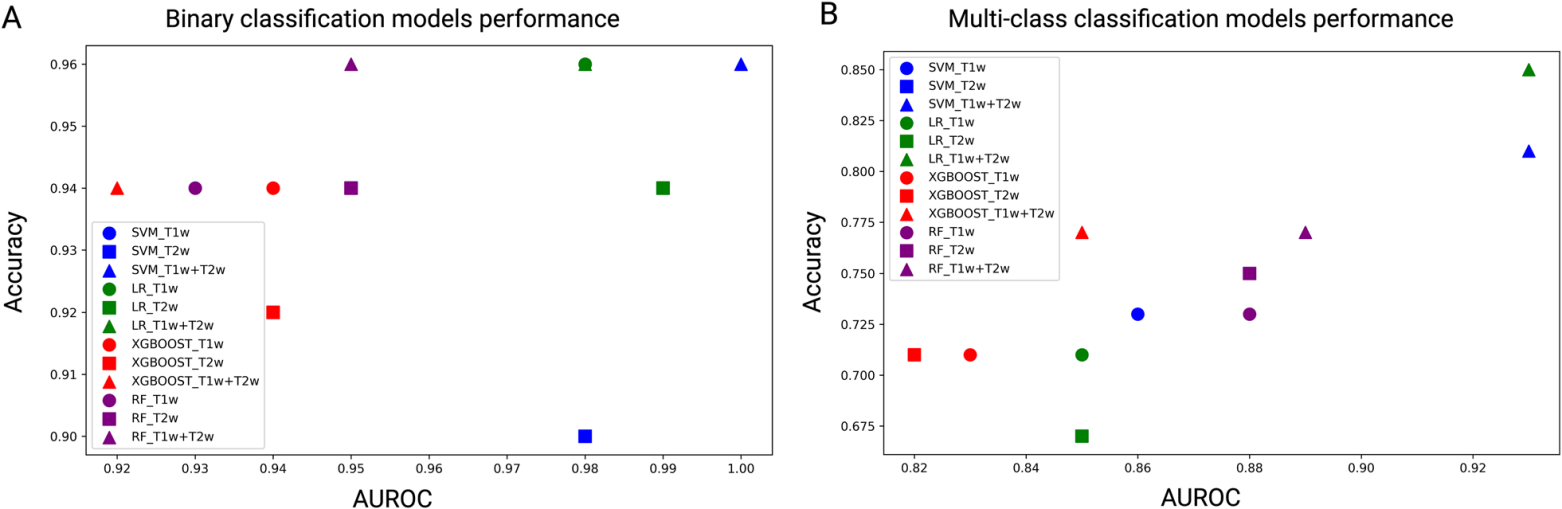
Performance of binary and multi-class classification models in HCC treatment response. **A** illustrates the performance of binary classification models, with each point representing a different machine learning model applied to T1wT1w, T2wT2w, or combined T1w + T2w MRI datasets. The x-axis indicates the AUROC and the y-axis shows the accuracy of each model. The symbols correspond to different models: squares for SVM, circles for LR, triangles for RF, and diamonds for XGBoost. **B** displays the performance of multi-class classification models, using the same symbol

Given the relatively constrained dataset size, to counteract potential model overfitting while maximizing the utility of our data, we performed LOOCV on each model. The evaluation of our model involved the calculation of (ROC) curves and the subsequent reporting of metrics such as AUROC, sensitivity, specificity, accuracy, and recall. For therapeutic prediction, four classification models, including SVM, XGBoost, LR and RF were developed, using the T1w, T2w, and combined T1w + T2w MRI data to differentiate between the control and treatment groups (NK IHA, sorafenib administration and combination therapy). The features identified through RFECV are shown in Table 1. The ROC curves, indicative of the predictive capacities of the four distinct models and metrics of each model, are presented in Figs. 3A–C and 4A, with detailed performance metrics in Additional file 1: Table S1, notably, SVM model utilizing the integrated T1w + T2w data emerged as the premier model, achieved an AUC of the ROC at 1.00, an accuracy rate of 96%, a sensitivity rate of 100%, a specificity rate of 83%, a precision rate of 97% and a recall rate of 96% in discerning treated from untreated HCC cases.

### Evaluation of the multi-class classification models

In clinical practice, binary classifications are often inadequate for diagnosing or forecasting treatment outcomes, underscoring the importance of multi-class models capable of discerning intricate treatment results. Our multi-class treatment outcomes classification models were based on five key features (Table 1). Among the evaluated models, RF model exhibited optimal results, obtained an AUC of the ROC at 0.93, an accuracy rate of 85%, a sensitivity rate of 85%, a specificity rate of 95%, a precision rate of 86% and a recall rate of 85% across four treatment groups: control, NK cells, sorafenib, and combination therapy. Comprehensive ROC curves and performance metrics for each model are illustrated in Figs. 3D–F and 4B, with detailed model performance provided in Additional file 1: Table S2. Notably, the combined T1w + T2w MRI model outperformed individual T1w and T2w MRI models.

### Correlation assessment of MRI features with histological tumor biomarkers

We established regression models to validate the relationship between histological tumor biomarkers and MRI radiomics features, evaluating them through RMSE and the Spearman correlation coefficient during leave-one-out cross-validation. Each biomarker was assessed using three distinct linear regression models corresponding to T1w, T2w, and combined T1w + T2w MRI datasets.

**Fig. 5:**
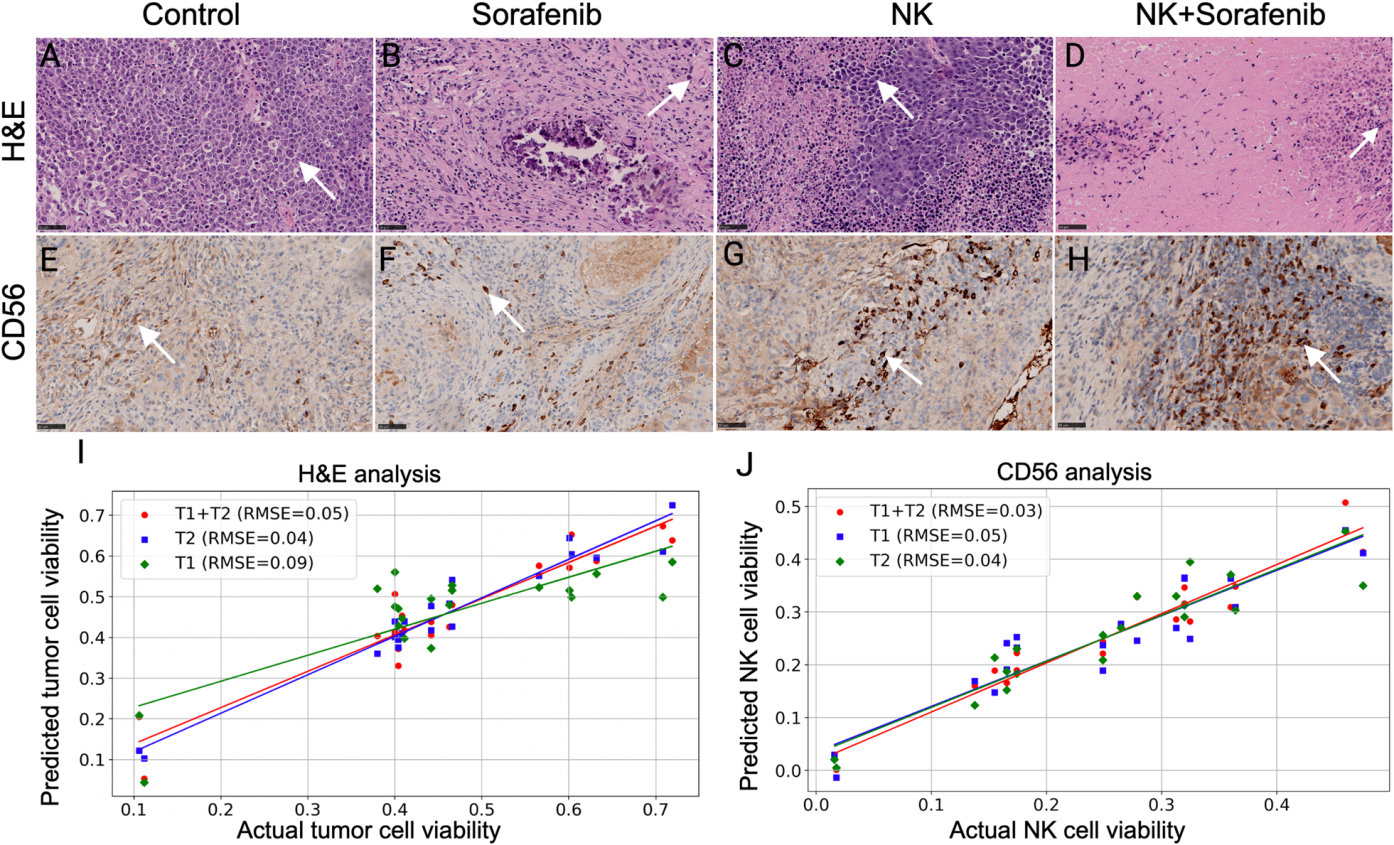
Histological Analysis of HCC Treatments with H&E and CD56 Staining. Arrow shows the tumor cell (dark pink) and NK cell (dark brown). **A**–**D** H&E-stained histological sections revealing the tumor cell viability for the (**A**) control group, (**B**) sorafenib treatment, (**C**) NK cell IHA treatment and (**D**) combination therapy. **E**–**H** CD56-stained sections highlighting NK cell activity for the (**E**) control group, (**F**) sorafenib treatment, (**G**) NK cell IHA treatment, and (**H**) combination therapy. **I**, **J** Correlation curves between MRI features and both (**I**) H&E and (**J**) CD56 histological biomarkers

H&E-stained histological images showed a significant difference in tumor cell viability between four treatment groups (Fig. 5A–D). All treatment groups—NK cell infusion, sorafenib, and their combination—substantially reduced tumor cell counts compared to the control (p < 0.05), with the combination therapy proving more effective than either treatment alone (p < 0.05). Notably, there was no significant difference in tumor cell reduction between the NK cell infusion and sorafenib groups, suggesting comparable efficacy. A multivariable analysis of these H&E-stained sections identified five key features, which informed the construction of regression models associated with tumor cell count. The T1w MRI model showcased an RMSE of 0.09 and a correlation coefficient of 0.92. The T2w MRI model achieved an RMSE of 0.04 and a correlation coefficient of 0.91, while the T1w + T2w MRI model yielded an RMSE of 0.05 with a correlation coefficient of 0.90 (Fig. 5I).

Inspection of CD56^+^-stained histological images discerned a significant variation in NK cell activity across the same four treatment groups (Fig. 5E–H). An increased presence of NK cells was significantly observed in the combination therapy group compared to the single-treatment and control groups (p < 0.05). Furthermore, NK cell immunotherapy induced a more pronounced NK cell migration compared to sorafenib treatment (p < 0.05), with both groups exhibiting a significant increase in NK cells relative to the control group (p < 0.05). For the CD56^+^ multivariable analysis, the models based on five chosen features, reported RMSE values of 0.05, 0.04, and 0.03 for T1w, T2w, and T1w + T2w MRI datasets, respectively, with corresponding correlation coefficients of 0.89, 0.92, and 0.94 (Fig. 5J).

## Discussion

In our study, we investigated the latent potential of radiomics features derived from MRI texture data to develop both binary and multi-class classification models for assessing treatment outcomes of HCC. The generated classification model demonstrated that the combination of T1w and T2w MRI texture features may serve as noninvasive biomarkers to predict treatment outcomes for NK cells, Sorafenib and their combination in SD rats model of HCC.

In recent years, the potential of MRI-based assessments for HCC has been explored in several studies [16, 21–23]. In an early study, Zhenyu developed and validated a radiomics model that leveraged MRI radiomic features to evaluate pathologic complete response (pCR) in LARC patients [22]. These findings suggest that MRI radiomic features might serve as noninvasive biomarkers for post-treatment predictors. Another study demonstrated MRI texture features can serve as imaging biomarkers for early therapeutic response detection post-DC vaccination in the KPC mouse model of PDAC and characterize the tumor microenvironment consistent with histological analysis [16]. Recently, a study highlighted that a quantitative analysis of MRI textures could distinguish the ablation zone following IRE ablation in animal models [21]. However, research regarding the prediction of multiple HCC treatment outcomes with high accuracy remains limited. Furthermore, conventional imaging biomarkers such as iRECIST [12], imRECIST, and irRC, which rely on changes in tumor size, are not always reliable in detecting novel immunotherapy responses [14, 24, 25]. Their limitations stem from not accounting for tumor heterogeneity and the potential inaccuracies introduced by pseudoprogression and mixed response patterns.

To overcome these challenges, we initially excluded features related to tumor size during the feature selection process. Subsequently, we developed statistical learning models utilizing texture MRI data and compared the performance of binary (treatment vs. control) and multi-class classifications across various HCC treatment methods, including NK cell IHA delivery, Sorafenib administration, and combination therapy. Our findings underscore that classification models based on T1w + T2w MRI texture features can effectively discern different HCC treatment outcomes. Notably, the classifier attained an AUC of 1.00 in binary classification and an AUC of 0.93 when distinguishing among the four treatment effects.

There are several limitations to our study. First, while our subject cohort is relatively limited in size, it aligns with the sample sizes of other preclinical studies. Moreover, techniques like SVM are especially effective for smaller datasets. To counterbalance this limitation, we employed leave-one-out cross-validation, fully utilizing the available data. Consequently, our study serves as an initial in vivo investigation, laying a foundation for subsequent evaluations on clinical datasets. Secondly, the manual segmentation of the tumor’s region of interest (ROI) introduces potential biases and is labor-intensive. Subsequent research might prioritize automated tumor segmentation or delve into multi-task approaches that both segment the tumor region and perform classification in tandem.

Future advancements in multi-class classification models, as evidenced by our research, may pave the way for improved decision-making processes in HCC therapy. Whereas traditional binary classification models are confined to distinguishing between the mere presence or absence of a treatment response, multi-class classifiers can detect subtle differences in treatment outcomes. This capability could assist clinicians in determining the most efficacious treatment regimens. In the future, these models could be refined through the integration of larger and more diverse datasets, improving their predictive accuracy and robustness. Additionally, the implementation of deep learning algorithms could further enhance the model's capability to analyze complex patterns within MRI data. Ultimately, these improvements will aim to provide a more personalized treatment strategy for patients to individual response profiles. As we continue to refine these models, we envision their application extending beyond HCC to other malignancies, leveraging MRI radiomics as a cornerstone for precision oncology.

## Conclusions

Our study has emphasized the considerable potential of texture-based MRI features in evaluating various treatment outcomes for HCC. By excluding tumor size features, we were able to address associated challenges, enhancing the accuracy of our results. The MRI-based radiomics model introduced herein holds promise as a noninvasive predictor, offering insights that could guide clinicians in making informed treatment decisions for HCC patients.

### Supplementary Information

**Additional file 1: Table S1.** Performance of binary classifiers of treatment vs control group. **Table S2.** performance of multi-class classifiers of different treatment outcomes.

## Data Availability

The datasets used and/or analysed during the current study are available from the corresponding author on reasonable request.

## Abbreviations

AUC: Area under the curve
AUROC: Area under the receiver operating characteristic
HCC: Hepatocellular carcinoma
IHA: Intrahepatic arterial
LR: Linear regression
MRI: Magnetic resonance imaging
RFECV: Recursive feature elimination with cross-validation
ROC: Receiver operating characteristic curve
RF: Random forest
SVM: Support vector machine

## References

[1] Sung H, Ferlay J, Siegel RL, Laversanne M, Soerjomataram I, Jemal A (2021). Global cancer statistics 2020: GLOBOCAN estimates of incidence and mortality worldwide for 36 cancers in 185 countries. CA Cancer J Clin.

[2] European Association for the Study of the Liver (2018). European association for the study of the L EASL clinical practice guidelines: management of hepatocellular carcinoma. J Hepatol.

[3] Llovet JM, Real MI, Montana X, Planas R, Coll S, Aponte J (2002). Arterial embolisation or chemoembolisation versus symptomatic treatment in patients with unresectable hepatocellular carcinoma: a randomised controlled trial. Lancet.

[4] Llovet JM, Ricci S, Mazzaferro V, Hilgard P, Gane E, Blanc JF (2008). Sorafenib in advanced hepatocellular carcinoma. N Engl J Med.

[5] Yang J, Eresen A, Scotti A, Cai K, Zhang Z (2021). Combination of NK-based immunotherapy and sorafenib against hepatocellular carcinoma. Am J Cancer Res.

[6] Cheng AL, Kang YK, Chen Z, Tsao CJ, Qin S, Kim JS (2009). Efficacy and safety of sorafenib in patients in the Asia-Pacific region with advanced hepatocellular carcinoma: a phase III randomised, double-blind, placebo-controlled trial. Lancet Oncol.

[7] Rezvani K, Rouce RH (2015). The application of natural killer cell immunotherapy for the treatment of cancer. Front Immunol.

[8] Tonn T, Schwabe D, Klingemann HG, Becker S, Esser R, Koehl U (2013). Treatment of patients with advanced cancer with the natural killer cell line NK-92. Cytotherapy.

[9] Guillerey C, Huntington ND, Smyth MJ (2016). Targeting natural killer cells in cancer immunotherapy. Nat Immunol.

[10] Shen YC, Hsu C, Cheng AL (2010). Molecular targeted therapy for advanced hepatocellular carcinoma: current status and future perspectives. J Gastroenterol.

[11] Sprinzl MF, Reisinger F, Puschnik A, Ringelhan M, Ackermann K, Hartmann D (2013). Sorafenib perpetuates cellular anticancer effector functions by modulating the crosstalk between macrophages and natural killer cells. Hepatology.

[12] Seymour L, Bogaerts J, Perrone A, Ford R, Schwartz LH, Mandrekar S (2017). iRECIST: guidelines for response criteria for use in trials testing immunotherapeutics. Lancet Oncol.

[13] Chiou VL, Burotto M (2015). Pseudoprogression and immune-related response in solid tumors. J Clin Oncol.

[14] Gerwing M, Herrmann K, Helfen A, Schliemann C, Berdel WE, Eisenblatter M (2019). The beginning of the end for conventional RECIST—novel therapies require novel imaging approaches. Nat Rev Clin Oncol.

[15] Lubner MG, Smith AD, Sandrasegaran K, Sahani DV, Pickhardt PJ (2017). CT texture analysis: definitions, applications, biologic correlates, and challenges. Radiographics.

[16] Eresen A, Yang J, Shangguan J, Li Y, Hu S, Sun C (2020). MRI radiomics for early prediction of response to vaccine therapy in a transgenic mouse model of pancreatic ductal adenocarcinoma. J Transl Med.

[17] Zhuang Z, Liu Z, Li J, Wang X, Xie P, Xiong F (2021). Radiomic signature of the FOWARC trial predicts pathological response to neoadjuvant treatment in rectal cancer. J Transl Med.

[18] Sheu AY, Zhang Z, Omary RA, Larson AC (2013). Invasive catheterization of the hepatic artery for preclinical investigation of liver-directed therapies in rodent models of liver cancer. Am J Transl Res.

[19] Yushkevich PA, Yang G, Gerig G (2016). ITK-SNAP: an interactive tool for semi-automatic segmentation of multi-modality biomedical images. Annu Int Conf IEEE Eng Med Biol Soc.

[20] van Griethuysen JJM, Fedorov A, Parmar C, Hosny A, Aucoin N, Narayan V (2017). Computational radiomics system to decode the radiographic phenotype. Cancer Res.

[21] Eresen A, Sun C, Zhou K, Shangguan J, Wang B, Pan L (2022). Early differentiation of irreversible electroporation ablation regions with radiomics features of conventional MRI. Acad Radiol.

[22] Liu Z, Zhang XY, Shi YJ, Wang L, Zhu HT, Tang Z (2017). Radiomics analysis for evaluation of pathological complete response to neoadjuvant chemoradiotherapy in locally advanced rectal cancer. Clin Cancer Res.

[23] Ren J, Qi M, Yuan Y, Duan S, Tao X (2020). Machine learning-based MRI texture analysis to predict the histologic grade of oral squamous cell carcinoma. AJR Am J Roentgenol.

[24] Marciscano AE, Thorek DLJ (2018). Role of noninvasive molecular imaging in determining response. Adv Radiat Oncol.

[25] Hodi FS, Ballinger M, Lyons B, Soria JC, Nishino M, Tabernero J (2018). Immune-modified response evaluation criteria in solid tumors (imRECIST): refining guidelines to assess the clinical benefit of cancer immunotherapy. J Clin Oncol.

