# Supplementary material for: MRI radiomics to monitor therapeutic outcome of sorafenib plus IHA transcatheter NK cell combination therapy in hepatocellular carcinoma
Source: J Transl Med. 2024 Jan 19;22:76. doi: 10.1186/s12967-024-04873-w (PMC10797785; doi:10.1186/s12967-024-04873-w)
Supplement: Supplementary file 1 — Additional file 1: Table S1. Performance of binary classifiers of treatment vs control group. Table S2. performance of multi-class classifiers of different treatment outcomes. [file 12967_2024_4873_MOESM1_ESM.docx]

**Table S1.** Performance of Binary Classifiers of Treatment vs Control Group.

| Dataset | Models | AUROC | Accuracy (%) | Sensitivity (%) | Specificity (%) | Precision (%) | Recall (%) |
| --- | --- | --- | --- | --- | --- | --- | --- |
| T1w | SVM | 0.99 | 94 | 100 | 75 | 95 | 94 |
|  | XGBoost | 0.94 | 94 | 97 | 83 | 94 | 94 |
|  | RF | 0.93 | 94 | 100 | 75 | 95 | 94 |
|  | LR | ­0.98 | 96 | 100 | 83 | 97 | 96 |
|  | | | | | | | |
| T2w | SVM | 0.98 | 90 | 100 | 58 | 94 | 90 |
|  | XGBoost | 0.94 | 92 | 97 | 75 | 93 | 92 |
|  | RF | 0.95 | 94 | 100 | 75 | 95 | 94 |
|  | LR | 0.99 | 94 | 100 | 75 | 95 | 94 |
|  | | | | | | | |
| T1w+T2w | **SVM** | **1.00** | **96** | **100** | **83** | **97** | **96** |
|  | XGBoost | 0.92 | 94 | 97 | 83 | 94 | 94 |
|  | RF | 0.95 | 96 | 100 | 83 | 97 | 96 |
|  | LR | 0.98 | 96 | 83 | 100 | 97 | 96 |

**Table S2.** Performance of Multi-Class Classifiers of Different Treatment Outcomes.

| Dataset | Models | AUROC | Accuracy (%) | Sensitivity (%) | Specificity (%) | Precision (%) | Recall (%) |
| --- | --- | --- | --- | --- | --- | --- | --- |
| T1w | SVM | 0.86 | 73 | 73 | 91 | 74 | 73 |
|  | XGBoost | 0.83 | 71 | 71 | 90 | 73 | 71 |
|  | RF | 0.88 | 73 | 73 | 91 | 73 | 73 |
|  | LR | 0.85 | 71 | 71 | 90 | 74 | 71 |
|  | | | | | | | |
| T2w | SVM | 0.85 | 67 | 62 | 80 | 71 | 67 |
|  | XGBoost | 0.82 | 71 | 70 | 83 | 71 | 71 |
|  | RF | 0.88 | 75 | 70 | 85 | 79 | 75 |
|  | LR | 0.85 | 67 | 62 | 80 | 71 | 67 |
|  | | | | | | | |
| T1w+T2w | SVM | 0.93 | 81 | 81 | 93 | 83 | 81 |
|  | XGBoost | 0.85 | 77 | 67 | 89 | 71 | 67 |
|  | RF | 0.89 | 77 | 77 | 92 | 78 | 77 |
|  | **LR** | **0.93** | **85** | **85** | **95** | **86** | **85** |
